# Buyang Huanwu Decoction improves energy metabolism disorders after cerebral ischemia–reperfusion by regulating the SIRT1/AMPK signaling pathway to promote glycolysis and the tricarboxylic acid cycle

**DOI:** 10.1186/s13020-025-01163-5

**Published:** 2025-07-07

**Authors:** Zhongji Hu, Nujiao Deng, Yanling Li, Yang Bai, Xiao Lan, Tingting Xiong, Huang Ding, Xiaodan Liu, Changqing Deng

**Affiliations:** 1 Key Laboratory of Hunan Province for Integrated Traditional Chinese and Western Medicine on Prevention and Treatment of Cardio-Cerebral Diseases, Hunan University of Chinese Medicine, Changsha, 410208 China; 2 The First Hospital of Hunan University of Chinese Medicine, Changsha, 410007 China

**Keywords:** Qi deficiency and blood stasis syndrome, Cerebral ischemia reperfusion injury, Buyang Huanwu Decoction, Energy metabolism, Metabolomics, Glycolysis, Tricarboxylic acid cycle

## Abstract

**Background:**

Buyang Huanwu Decoction (BYHWD), a traditional Chinese medicine formula for cerebral infarction, exerts neuroprotective effects by enhancing cerebral energy metabolism, yet its precise mechanisms remain elusive.

**Objective:**

To explore the effects of BYHWD on improving cerebral ischemia–reperfusion injury (CIRI) with Qi deficiency and blood stasis syndrome from an energy metabolism perspective and verify it through experiments.

**Methods:**

A rat model of CIRI with Qi deficiency and blood stasis syndrome was established and intervened with BYHWD. The therapeutic effect of BYHWD was evaluated using Longa score, Qi deficiency and blood stasis syndrome score, pathological staining, and colorimetric assays. Untargeted metabolomics was used to identify differential metabolites and regulatory mechanisms, and in vivo and in vitro models were constructed for validation.

**Results:**

BYHWD ameliorated neurological deficits and Qi deficiency and blood stasis syndrome in rats, reduced brain pathology, and increased energy substances. Untargeted metabolomics analysis suggested BYHWD enhanced cerebral energy metabolism via nicotinate and nicotinamide metabolism and AMPK signaling, involving SIRT1/AMPK regulation and promotion of glycolysis and the tricarboxylic acid (TCA) cycle. Validation experiments showed BYHWD activated the SIRT1/AMPK signaling pathway in brain tissue, promoting glucose uptake and enhancing the expression of proteins related to glycolysis, mitochondrial biogenesis, and the TCA cycle. Similar results were observed in HT22 cells subjected to oxygen–glucose deprivation/reperfusion (OGD/R).

**Conclusion:**

BYHWD improved cerebral energy metabolic disorders by activating the SIRT1/AMPK signaling pathway, thereby enhancing glycolytic capacity and TCA cycle capacity. This study elucidated the mechanisms of BYHWD and provided a theoretical basis for its rational application.

## Introduction

Cerebral infarction, characterized by its high incidence, mortality, and disability rates, is a severe cerebrovascular disease that critically threatens human life [1]. Despite advances in thrombolytic and interventional therapies in recent years, secondary injuries caused by post-reperfusion further exacerbate cerebral damage, impairing neurological recovery and prognosis. Consequently, enhancing strategies to prevent and treat cerebral ischemia–reperfusion injury (CIRI) is pivotal for improving post-ischemic neurological restoration.

Energy serves as the cornerstone for maintaining normal brain physiological activities [2]. Although the brain constitutes merely 2% of total body weight, it consumes 20% of the body’s oxygen and energy, making it the most energy-demanding organ and highly sensitive to disruptions in energy metabolism [3]. Following cerebral ischemia, stored adenosine triphosphate (ATP) is depleted within 5–7 min. Since neuronal cells cannot actively regenerate ATP, energy metabolism dysfunction rapidly occurs [4], triggering severe ischemic cascades that worsen pathological brain injury [5]. Impaired energy metabolism persists throughout the ischemia/reperfusion process, playing a central role in its initiation, progression, and aggravation. Therefore, ameliorating energy metabolism dysfunction is a crucial therapeutic approach to mitigate post-ischemic damage.

Traditional Chinese Medicine (TCM) posits that Qi deficiency and blood stasis form the core pathogenesis of cerebral ischemia, with benefiting Qi and activating blood circulation as the primary treatment principle. The Buyang Huanwu Decoction (BYHWD), a classic TCM formula for replenishing Qi and resolving blood stasis, has shown significant clinical efficacy in treating cerebral infarction by promoting neurological recovery and improving cerebral function [6, 7]. Preclinical studies demonstrate that BYHWD alleviates CIRI-induced brain injury through multiple mechanisms, including suppression of oxidative stress and inflammation [8–10], regulation of mitochondrial quality control [11, 12], and enhancement of angiogenesis [13]. However, research on BYHWD’s role in modulating energy metabolism dysfunction during CIRI remains limited. Although Chen et al. utilized proteomics and high-throughput sequencing to suggest BYHWD’s involvement in CIRI energy metabolism regulation [14, 15], its precise molecular targets and mechanisms remain unclear.

Building on this foundation, this study aims to establish a rat model of CIRI with Qi deficiency and blood stasis syndrome that integrates disease and TCM syndrome characteristics, evaluate BYHWD’s pharmacological effects, and employ untargeted metabolomics to identify its key targets and pathways in ameliorating energy metabolism dysfunction. Subsequent validation using in vivo and in vitro models will further elucidate the mechanisms by which BYHWD improves energy metabolism in cerebral infarction with Qi deficiency and blood stasis syndrome.

## Materials and methods

### Experimental animals

A total of 120 adult male Sprague Dawley (SD) rats of uniform age (6–8 weeks) and weight (180–220 g) were obtained from Hunan Sleckjingda Co., Ltd., license number: SCXK (Xiang) 2019-0004. Experimental unit usage license number: SYXK (Xiang) 2019-0009. Quality certificate number: 43072723110205484. The animals were kept in the Experimental Animal Center of Hunan University of Traditional Chinese Medicine, with a temperature of 24–26 °C and humidity of 50–70%. They were fed with sterilized feed, and the rats were free to drink and feed. This experiment was reviewed and approved by the Experimental Animal Ethics Committee of Hunan University of Traditional Chinese Medicine (Ethics Review Batch Number: LLBH-202302200001).

### Experimental cells

Mouse hippocampal neuronal HT22 cells (Batch No.: CL-0697) were obtained from Procell Company.

### Experimental drugs

The components of BYHWD and their related information were shown in Table 1. All herbal materials were purchased from Hunan Chunkehui Traditional Chinese Medicine Company, and include *Radix Astragalus* (Batch No. CP-A069AA), *Radix Paeoniae rubra* (Batch No. CP-A025), *Rhizoma Chuanxiong* (Batch No. CP-A031), *Radix Angelicae sinensis* (Batch No. CP-A039A), *Lumbricus* (Batch No. CP-H029a), *Semen Persicae* (Batch No. CP-B091a), *Flos Carthami* (Batch No. CP-D004). All herbal materials were sourced from their primary production areas, the voucher specimen was kept in the Key Laboratory of Hunan Province for Integrated Traditional Chinese and Western Medicine on Prevention and Treatment of Cardio-Cerebral Diseases in Hunan University of Chinese Medicine. Butylphthalide soft capsules (Batch No. H20050299) were obtained from Enbipu Pharmaceutical Co., Ltd. of Shijiazhuang Pharmaceutical Group. Butylphthalide, a novel anti-cerebral ischemia drug, can improve cerebral circulation and promote neurological recovery [16]. It was prepared as a suspension with double-distilled water before use. 5-Aminoimidazole-4 -carboxamide ribonucleotide (AICAR, Batch No. 2627-69-2) was purchased from MCE Company. AICAR, an adenosine analog, activates the AMPK signaling pathway and regulates energy metabolism [17]. It was dissolved in DMSO to make a 1 mmol/L solution for use.

**Table 1:** An overview of the BYHWD

TCM name	Latin name	Location	Origin	Contents (g)
Huangqi	*Radix Astragali*	Inner Mongolia	*Astragalus membranaceus (Fisch.) Bunge, Leguminosae*	60
Chishao	*Radix Paeoniae rubra*	Sichuan	*Paeonia lactiflora Pall., Paeoniaceae*	6
Chuanxiong	*Rhizoma Chuanxiong*	Sichuan	*Ligusticum chuanxiong Hort., Umbelliferae*	6
Danggui	*Radix Angelicae sinensis*	Gansu	*Angelica sinensis (Oliv.) Diels, Umbelliferae*	9
Dilong	*Lumbricus*	Guangxi	*Pheretima aspergillum (E. Perrier), Megascolecidae*	9
Taoren	*Semen Persicae*	Shandong	*Prunus persica (L.) Batsch, Rosaceae*	9
Honghua	*Flos Carthami*	Xinjiang	*Carthamus tinctorius L., Compositae*	9

### Main reagents

Hematoxylin–eosin staining kit (Batch No. G1120) and Nissl staining kit (Batch No. G1434) were purchased from Solarbio Co. 2,3,5-Triphenyltetrazolium chloride (Batch No. T8877-10G) was obtained from Sigma Co. 0.34 mm middle cerebral artery occlusion (MCAO) filament (Batch No. 3040-A4) was purchased from Beijing Cinontech Co. RIPA lysis buffer (Batch No. CW2333S), protease inhibitor cocktail (Batch No. CW2200S), and phosphatase inhibitor cocktail (Batch No. CW2333S) were obtained from Cwbiotech Co. Enhanced chemiluminescence (ECL) kit (Batch No. SQ101) was purchased from Shanghai Ya Mei Bio-Pharmaceutical Technology Co. Tri-color prestained protein marker (8–180 kDa) (Batch No. 17001) was obtained from Zenbio Co. Methanol (Batch No. A456-4), formic acid (Batch No. A117-50), and ammonium acetate (Batch No. A114-50, pH 9.0) were purchased from Thermo Fisher Co. Fetal bovine serum (Batch No. 164210-50) and high-glucose (dulbecco's modified eagle medium) DMEM (Batch No. PM150210) were obtained from Procell Bio Co. Dimethyl sulfoxide (Batch No. 67-68-5) was purchased from MCE Co. Hexokinase (HK) activity assay kit (Batch No. E-BC-K610-M), phosphofructokinase (PFK) activity assay kit (Batch No. E-BC-K612-M), pyruvate kinase (PK) activity assay kit (Batch No. E-BC-K611-M), citrate synthase (CS) activity assay kit (Batch No. E-BC-K178-M), nicotinamide adenine dinucleotide-isocitrate dehydrogenase (NAD-IDH) activity assay kit (Batch No. E-BC-K651-M), α-ketoglutarate dehydrogenase (α-KGDH) activity assay kit (Batch No. E-BC-K083-M), adenosine triphosphate (ATP) colorimetric assay kit (Batch No. E-BC-F002), adenosine diphosphate (ADP) fluorescent assay kit (Batch No. E-BC-F009), mitochondrial complex I, II, III, IV activity assay kits (Batch Nos. E-BC-K149-M, E-BC-K150-M, E-BC-K151-M, E-BC-K152-M), extracellular acidification rate (ECAR) assay kit (Batch No. E-BC-F069), cellular oxygen consumption rate (OCR) assay kit (Batch No. E-BC-F068), CCK-8 assay kit (Batch No. E-CK-A362), lactate dehydrogenase (LDH) activity assay kit (Batch No. E-BC-K771-M), and BCA protein concentration assay kit (Batch No. E-BC-K318-M) were all purchased from Elabscience Biotechnology Co. ADP ELISA kit (Batch No. AF2923-A) and nicotinamide adenine dinucleotide (NADH) ELISA kit (Batch No. AF21266-A) were obtained from Afantibody Co. Nicotinamide phosphoribosyltransferase (NAMPT) rabbit anti-rat polyclonal antibody (Batch No. 11776-1-AP), AMP-activated protein kinase (AMPK) rabbit anti-rat polyclonal antibody (Batch No. 18167-1-AP), phosphofructokinase M (PFKM) rabbit anti-rat polyclonal antibody (Batch No. 55028-1-AP), citrate synthase (CS) rabbit anti-rat polyclonal antibody (Batch No. 16131-1-AP), 2-oxoglutarate dehydrogenase (OGDH) mouse anti-rat monoclonal antibody (Batch No. 66285-1-Ig), isocitrate dehydrogenase 3 (NAD^+^) alpha (IDH3A) rabbit anti-rat polyclonal antibody (Batch No. 15909-1-AP), and beta-actin (β-actin) rabbit anti-rat monoclonal antibody (Batch No. 66009-1-Ig) were all purchased from Proteintech Co. Silent information regulator 1 (SIRT1) rabbit anti-rat monoclonal antibody (Batch No. R25721), phospho-AMP-activated protein kinase (p-AMPK) (Thr172) rabbit anti-rat monoclonal antibody (Batch No. R381164), peroxisome proliferator-activated receptor gamma coactivator 1-alpha (PGC-1α) rabbit anti-rat monoclonal antibody (Batch No. 381615), nuclear respiratory factor 1 (NRF1) rabbit anti-rat monoclonal antibody (Batch No. R25184), beta-tubulin (β-tubulin) rabbit anti-rat polyclonal antibody (Batch No. 390628), glucose transporter 4 (GLUT4) rabbit anti-rat polyclonal antibody (Batch No. 347063), hexokinase II (HKII) rabbit anti-rat monoclonal antibody (Batch No. R24552), and pyruvate kinase M (PKM) rabbit anti-rat polyclonal antibody (Batch No. 321004) were all obtained from Zenbio Co. Mitochondrial transcription factor A (TFAM) rabbit anti-rat polyclonal antibody (Batch No. AF0531) was purchased from Affinity Co. Goat anti-rabbit IgG (H + L)-HRP (Batch No. db10003) and goat anti-mouse IgG (H + L)-HRP (Batch No. db10002) were obtained from Diagbio Co.

## Experimental methods

### Investigation of BYHWD in ameliorating energy metabolism dysfunction following CIRI with Qi deficiency and blood stasis syndrome

#### Drug preparation

BYHWD was formulated with the following composition: *Radix Astragalus* (Huangqi) 60 g, *Radix Paeoniae rubra* (Chishao) 6 g, *Rhizoma Chuanxiong* (Chuanxiong) 6 g, *Radix Angelicae sinensis* (Danggui) 9 g, *Lumbricus* (Dilong) 9 g, *Semen Persicae* (Taoren) 9 g, *Safflower* (Honghua) 9 g. Crude herbs were weighed, fragmented, and subjected to reflux extraction in triplicate. For the initial extraction, an eightfold volume of water was added, followed by 2 h of gentle boiling. The decoction was cooled and filtered. Subsequent extractions utilized sixfold volumes of water with 1-h boiling periods. All extracts were pooled, filtered through 0.45 μm membranes, and vacuum-concentrated under reduced pressure to a crude drug concentration of 1000 g/L. The concentrated solution was aliquoted and stored at – 20 °C until use.

The main effective components of BYHWD were identified by querying the 2020 edition of the Chinese Pharmacopoeia as *Hydroxysafflor yellow A, Amygdalin, Paeoniflorin, ferulic acid, Calycosin-7-O-beta-D-glucoside and Astragaloside IV*. Based on this, our research team employed UPLC-Q-TOF-MS/MS to analyze the in vitro and in vivo active components of BYHWD. The results showed that these key active components were detected in both the water extract of BYHWD and the drug-containing serum, and the relevant data are shown in Table 2.

**Table 2:** The main effective components of BYHWD

Effective components	Molecular formula	Molecular weight	Ionic mode	TR/min	Content mg/mL	Source
*Hydroxysafflor yellow A*	C27H32O16	612.169	M-H 611.1585	9.159	0.00043099	Honghua
*Amygdalin*	C20H27NO11	457.1584	M + NH 4475.1924	9.449	0.00012307	Taoren
*Paeoniflorin*	C23H28O11	480.1632	M + Na 503.1527	13.868	0.02427259	Chishao
*Ferulic acid*	C10H10O4	194.0579	M-H 193.0565	14.609	0.04830363	Huangqi, Honghua, Chuanxiong, Danggui, Taoren
*Calycosin-7-O-beta-D-glucoside*	C22H22O10	446.404	M + HCOO 491.4025	14.692	0.00177258	Huangqi
*Astragaloside IV*	C41H68O14	784.4559	M-H 783.4477	22.862	0.00407272	Huangqi

#### Animal model preparation

A rat model of exhaustive swimming for 21 days and MCAO for CIRI was established [18, 19]: rats were acclimatized and fed for 3 days, and then loaded according to the body weight of 4.0 ± 0.5%, and placed in the water with the water temperature of 20 ± 1 ℃ and forced to swim to the exhaustion (the judgment criterion was that the tip of the nose of the rat sank in the water 3 times continuously for more than 10 s each time, accompanied by the dysfunction of the swimming movement), once a day, for 21 days in a row. Model of focal CIRI was prepared the day after the exhaustion swim. Rats were anesthetized by intraperitoneal injection of 2.5% pentobarbital sodium (2 mL/kg), and the left common carotid artery, external carotid artery, and internal carotid artery were isolated. A small incision was made at the bifurcation of the left common carotid artery, and a wire was inserted into the left internal carotid artery until resistance was encountered, indicating successful occlusion of the middle cerebral artery. The filament was removed after 2 h of ischemia to restore blood perfusion, and the CIRI model was prepared, and the Longa neurological function score was performed after waking up [20], and a score of 1–3 was regarded as successful modeling.

#### Animal grouping and treatment

The adult daily dose of BYHWD is 1.8 g/kg. Using the rat—to—human conversion factor of 6.2 from the U.S. Food and Drug Administration, the daily dose for rats is calculated at 11.1 g/kg. Previous studies by our team found that BYHWD had a significant therapeutic effect on cerebral ischemia/reperfusion injury in rats when the crude drug concentration of BYHWD was 22.2 g/kg/day (equivalent to twice the recommended adult dose) [10, 18, 21]. Therefore, we used BYHWD crude drug concentration of 11.1 g/kg/day versus 22.2 g/kg/day as the administered dose for the study. The rats were randomly divided into sham operation (Sham) group, composite modeling (ES + I/R) group, BYHWD low dose (BYHWD-L) group, BYHWD high dose (BYHWD-H) group, and butylphthalide (NBP) group. The rat model of CIRI with Qi deficiency and blood stasis type was constructed by continuous forceful exhaustion swimming combined with MCAO in all groups except the Sham group. The BYHWD-L group, the BYHWD-H group and the NBP group were pre-administered one day prior to the MCAO operation, and the drug was continuously administered twice daily during the period of reperfusion. Equal volumes of saline were synchronously gavaged in the Sham group and the ES + I/R group and taken for testing after 48 h of reperfusion. Samples were taken for testing after 48 h of reperfusion.

#### Index detection

##### Neurological function deficit scoring (longa method) score

According to the Longa scoring method [20], neurological deficit scoring was performed immediately after modeling and 48 h after reperfusion: 0 points for no obvious neurological deficit; 1 point for mild deficit (right forepaw cannot extend); 2 points for moderate deficit (walking in a circle to the right); 3 points for severe deficit (falling to the right when walking); and 4 points for very severe deficit (unable to walk independently with loss of consciousness). Higher scores indicate more severe deficits, and the scores at 48 h after reperfusion were used for analysis.

##### Qi deficiency and blood stasis syndrome score

The scoring criteria for Qi deficiency syndrome was as follows [22]: ① Mental fatigue, with limbs exhibiting reduced activity (3 points); lethargy and drowsiness accompanied by mental fatigue (5 points); significantly decreased or absent antagonistic behavior (7 points). ② Hair becomes dry and lacks luster (3 points); hair mats and curls (5 points); hair is sparse and easily falls out (7 points). ③ Anorexia with mild diarrhea (3 points); anorexia with moderate diarrhea (5 points); anorexia with severe diarrhea or foul-smelling yellow-greenbrown stools (7 points). The scoring criteria for blood stasis syndrome was: ① Dark tongue color (3 points); purplish tongue (5 points). ② Eyeballs change from bright red to pale red (3 points); transition to dark red (5 points). ③ The skin color of the tail changes from pointed to root, appearing dull (3 points); slightly dark purple (5 points).The total score for Qi deficiency and blood stasis syndrome was obtained by summing the individual scores. A higher total score indicated more severe manifestations of Qi deficiency and blood stasis.

##### 2,3,5-triphenyltetrazolium chloride (TTC) staining for cerebral infarction and edema rates

After scoring, rats were anesthetized again with 2.5% pentobarbital sodium (2 mL/kg), and the whole brain was extracted and placed in phosphate buffer solution (PBS) at 4 ℃, and quickly transferred to -20 ℃ refrigerator for freezing for 30 min. The olfactory bulb, cerebellum and brainstem were removed, and the slices were sectioned in coronal position with a slice thickness of 2 mm. The slices were placed in 2% TTC solution and incubated at 37 °C for 30 min protected from light, and the slices were turned every 5 min to ensure full contact with the solution. The brain slices were then washed three times with PBS solution, fixed in 4% paraformaldehyde for 6 h and photographed. Image J software was used to analyze the infarcted area of the brain slices and calculate the cerebral infarction rate (cerebral infarction rate = total area of the infarcted area of 5 slices/total area of the brain slices of 5 slices × 100%) and the cerebral edema rate (cerebral edema rate = volume of infarcted side—volume of the infarcted area of the infarcted area of the brain/volume of the infarcted side of the brain × 100%).

##### Hematoxylin–Eosin (HE) staining for observing pathological changes in brain tissue

48 h after reperfusion, rats were perfused with 0.9% sodium chloride solution through the left ventricle until the liver turned grayish-white. The left brain was removed and fixed with 4% paraformaldehyde, routinely processed into paraffin sections, stained with HE, and observed under a microscope for pathological changes in the hippocampal region of the ischemic left brain. The cell damage rate was calculated using TissueFAXS Viewer software.

##### Nissl staining for observing the number of Nissl bodies in rat rrain tissue

Left brain paraffin sections were prepared as in HE staining, stained with Nissl, and observed under a microscope for neuronal morphology in the hippocampal region. The number of Nissl-positive cells was counted using TissueFAXS Viewer software for statistical analysis.

##### Colorimetric assay for measuring ATP, ADP, and NADH content in brain tissue

Rats were sacrificed 48 h after reperfusion, and the ischemic side brain tissue was collected. Brain tissue homogenate was prepared on ice with PBS, and protein concentration was measured. The levels of ATP, ADP, and NADH in brain tissue were measured by colorimetric assay according to the kit instructions.

##### Untargeted metabolomics for differential metabolite profiling in serum and brain tissue

After 48 h of reperfusion, rats were anesthetized, blood was collected from the abdominal aorta to separate serum, and ischemic brain tissue was harvested and stored at – 80 °C. Untargeted metabolomics analysis was performed using the ultra-high-performance liquid chromatography-tandem mass spectrometry (UHPLC-MS/MS) platform provided by Novogene Co., Ltd. (Beijing, China). Serum samples (100 μL) were placed in eppendorf tubes, resuspended in prechilled 80% methanol, and vortexed thoroughly. After incubation on ice for 5 min, the samples were centrifuged at 15,000 *g* and 4 °C for 20 min. The supernatant was diluted with LC–MS grade water to a final methanol concentration of 53%, transferred to a fresh eppendorf tube, and recentrifuged under identical conditions. The clarified supernatant was then injected into the LC–MS/MS system for analysis [23, 24]. For brain tissue samples (100 mg), tissue was pulverized in liquid nitrogen and homogenized in prechilled 80% methanol. Subsequent sample preparation is the same as serum sample preparation [25].

Untargeted metabolomics analysis used the Vanquish UHPLC chromatography system coupled with the Q Exactive^™^ HF-X mass spectrometer. The chromatographic conditions were as follows: Hypersil Gold C18 column (100 × 2.1 mm, 1.9 μm; column temperature 40 ℃; flow rate 0.2 mL/min). The mobile phase in positive ion mode was 0.1% formic acid aqueous solution (A)-methanol (B), and in negative ion mode, it was 5 mM ammonium acetate (pH 9.0, A)-methanol (B). The mass spectrometry scan range was set at m/z 100–1500, with data-dependent scanning in positive and negative ion modes. During data preprocessing, the raw files were imported into CD 3.1 software. Metabolites were screened by retention time and m/z, followed by peak alignment, peak extraction, and quantification using peak areas. Molecular formulas were predicted from molecular and fragment ions, and metabolites were identified by database matching. Blank samples were used to remove background interference. After data normalization and removal of compounds with CV > 30% in quality control (QC) samples, metabolite identification and relative quantification were achieved. Data processing was performed on a Linux system using R and Python programming languages. For statistical analysis, metabolites were annotated using KEGG, HMDB, and LIPIDMaps databases. Data were transformed with metaX software [26], and principal component analysis (PCA) and partial least squares discrimination analysis (PLS-DA) analyses were conducted to calculate VIP values. t-tests determined *P*-values and fold changes (FC). Differential metabolites were selected based on VIP > 1, *P* < 0.05, and FC ≥ 2 or ≤ 0.5. Finally, volcano plots, heatmaps, and bubble charts were created using R packages to analyze metabolite correlations and pathways.

##### Colorimetric assays for key enzyme activities

Brain homogenates were prepared as described in Sect. "Colorimetric assay for measuring ATP, ADP, and NADH content in brain tissue". The activities of glycolytic enzymes (HK, PFK, PK), tricarboxylic acid (TCA) cycle rate-limiting enzymes (CS, NAD-IDH, α-KGDH), and mitochondrial respiratory chain complexes I–IV were measured using colorimetric kits according to the manufacturer’s instructions.

##### Western blot analysis of protein expression of NAMPT, SIRT1, p-AMPK, AMPK, GLUT4, key glycolytic enzymes (HKII, PFKM, PKM), mitochondrial biogenesis-related proteins (PGC-1α, NRF1, TFAM), and key TCA cycle enzymes (CS, OGDH, IDH3a) in brain tissue

Brain tissue was collected from the injured side to prepare protein supernatant. A small portion was taken for protein concentration determination. Protein loading buffer was added to the remaining portion for protein denaturation. Electrophoresis was performed at 80 V constant voltage, followed by transfer at 200 mA constant current. After transfer, the PVDF membrane was blocked in 5% skimmed milk for 120 min. Then, the membrane was washed with TBST solution three times, each for 10 min. The membrane was incubated overnight at 4 °C with the following primary antibodies: NAMPT (1:1000), SIRT1 (1:500), p-AMPK (1:500), AMPK (1:1000), GLUT4 (1:500), HKII (1:5000), PFKM (1:5000), PKM (1:500), PGC-1α (1:500), NRF1 (1:3000), TFAM (1:500), CS (1:5000), IDH3A (1:5000), OGDH (1:5000), β-actin (1:5000), and Tubulin (1:5000). After primary antibody incubation, the membrane was washed with TBST solution three times, each for 10 min. The membrane was then incubated with the corresponding secondary antibodies: goat anti-rabbit (1:20000) or goat anti-mouse (1:20000), at room temperature for 90 min. The membrane was washed again with TBST solution three times, each for 10 min. The membrane was developed using ECL developing solution. The integral optical density (IOD) of the target proteins was measured using Image Lab image analysis software. The relative expression of the target protein was calculated as the ratio of its IOD to that of the internal reference protein.

### Study on BYHWD alleviating energy metabolism dysfunction in HT22 cells after OGD/R injury

#### Preparation of drug-containing serum

The preparation of BYHWD was as previously described. The drug solution was concentrated to 2 g/mL, and the gavage dose for rats was set at 5 times the equivalent dose (crude drug concentration of 11.1 g/kg/day). Specifically, for a 250 g rat, the daily gavage amount was approximately 14 g, with a daily gavage volume of 7 mL. 20 SD rats were taken and divided into blank serum group (*n* = 10) and drug-containing serum group (*n* = 10). Rats in the drug-containing serum group were gavaged twice daily with 3.5 mL of the drug solution for seven consecutive days. 4 h after the last gavage, the rats were anesthetized, blood was collected from the abdominal aorta, and then centrifuged to separate the serum. Complement in the serum was inactivated to prepare the drug-containing serum. The serum from each group of rats was mixed in equal amounts, aliquoted, and stored for later use. Rats in the blank serum group were gavaged with an equal amount of normal saline, and the other procedures were the same as those in the drug-containing serum group.

#### Cell grouping and interventions

The HT22 cell line, which retains basic neuronal functions and mimics pathological changes CIRI after OGD/R, was used as an ideal in vitro model for studying cerebral ischemia. The method for constructing the HT22 cell OGD/R model was as follows: HT22 cells were first cultured in high-glucose DMEM medium containing 10% fetal bovine serum (FBS) at 37 °C with 5% CO₂. Once fully adhered, the cells were trypsinized, centrifuged, resuspended, and counted. They were then seeded into 96-well plates at 2000 cells per well and cultured under the same conditions. After re-adhesion, the medium was replaced with glucose-free DMEM with 10% FBS, and cells were subjected to 4 h of OGD in a tri-gas incubator (37 °C, 94% N₂, 5% CO₂, 1% O₂). Subsequently, cells were transferred to a standard incubator with reoxygenation/reperfusion for 24 h after replacing the medium with normal high-glucose DMEM. Preliminary experiments confirmed that this protocol significantly reduced cell viability, achieving a 50–70% cell damage rate, establishing it as the optimal condition for constructing the OGD/R model.

HT22 cells were divided into six groups based on intervention: Control, OGD/R, BYHWD-L (2.5% drug-containing serum), BYHWD-M (5% drug-containing serum), BYHWD-H (10% drug-containing serum), and positive control AICAR (1 mmol/L). All groups were subjected to interventions with blank serum, drug-containing serum, and AICAR 24 h prior to OGD/R treatment, followed by OGD/R. The grouping and intervention are shown in Table 3.

**Table 3:** Cell grouping and intervention

Group	OGD/R	Culture condition
Control group	No	DMEM high-glucose medium with 10% fetal bovine serum
OGD/R group	Yes	DMEM high-glucose medium with 10% fetal bovine serum
BYHWD-L group	Yes	DMEM high-glucose medium with 2.5% drug-containing serum and 10% fetal bovine serum
BYHWD-M group	Yes	DMEM high-glucose medium with 2.5% drug-containing serum and 10% fetal bovine serum
BYHWD-H group	Yes	DMEM high-glucose medium with 2.5% drug-containing serum and 10% fetal bovine serum
AICAR group	Yes	DMEM high-glucose medium with 1 mmol/L AICAR and 10% fetal bovine serum

AICAR, an AMPK activator, promotes cellular metabolic regulation, improves energy metabolism, and has cytoprotective effects. Studies have confirmed that AICAR at a concentration of 1 mmol/L can effectively stimulate AMPK activation in HT22 cells, improving cellular energy metabolism [27]. Therefore, this concentration was chosen for intervention in this experiment.

#### Index detection

##### CCK-8 assay for cell viability

Cells were seeded in a 96-well plate and cultured until adherent. Subsequently, cells were treated in accordance with the experimental design. Then, 10 μL of CCK-8 reagent was added to each well, followed by incubation at 37 °C for 60 min. The absorbance (OD value) of each well was measured at 450 nm using a microplate reader, following the manufacturer's instructions. The OD value is positively correlated with cell proliferation or survival.

##### LDH assay for cell injury evaluation

Cells were treated as described in Sect. "Cell grouping and interventions". Cell culture medium was collected, centrifuged, and the supernatant was used for LDH quantification following the manufacturer's instructions.

##### ATP and ADP content assay in cells

Cells were cultured as described in Sect. "Cell grouping and interventions". A total of 2 × 10⁶ cells per group were collected for ATP and ADP detection following the manufacturer's instructions.

##### Fluorescence assay for extracellular acidification rate (ECAR) and oxygen consumption rate (OCR)

Cells were seeded in a 96-well black-bottom enzyme-labeled plate. Cells were treated as described in Sect. "Cell grouping and interventions". Fluorescent probe stock solutions were prepared, and the assay was performed following the manufacturer's instructions.

##### Western blot analysis of protein expression of NAMPT, SIRT1, p-AMPK, AMPK, GLUT4, key glycolytic enzymes (HKII, PFKM, PKM), mitochondrial biogenesis-related proteins (PGC-1α, NRF1, TFAM), and key TCA cycle enzymes (CS, OGDH, IDH3a) in cell

The cell culture flask was removed from the incubator, and the medium was discarded. After adding 1 mL of PBS, cells were collected using a cell scraper. Protein supernatant was prepared using protein lysis buffer. Subsequent steps followed the animal experimental protocol.

### Statistical analysis

All measured parameters were quantitative data expressed as mean ± SD. Experimental data were analyzed using statistical product and service solutions (SPSS) software. If the data met the assumptions of normality and homogeneity of variance, one-way analysis of variance (ANOVA) with least significant difference (LSD) post-hoc test was applied for intergroup comparisons. For data with heterogeneity of variance, the Kruskal–Wallis rank-sum test (non-parametric test) was used. *P*-value < 0.05 was considered statistically significant.

## Results

### BYHWD significantly reduces neurological deficits and pathological damage in CIRI rats with Qi deficiency and blood stasis syndrome

**Fig. 1:**
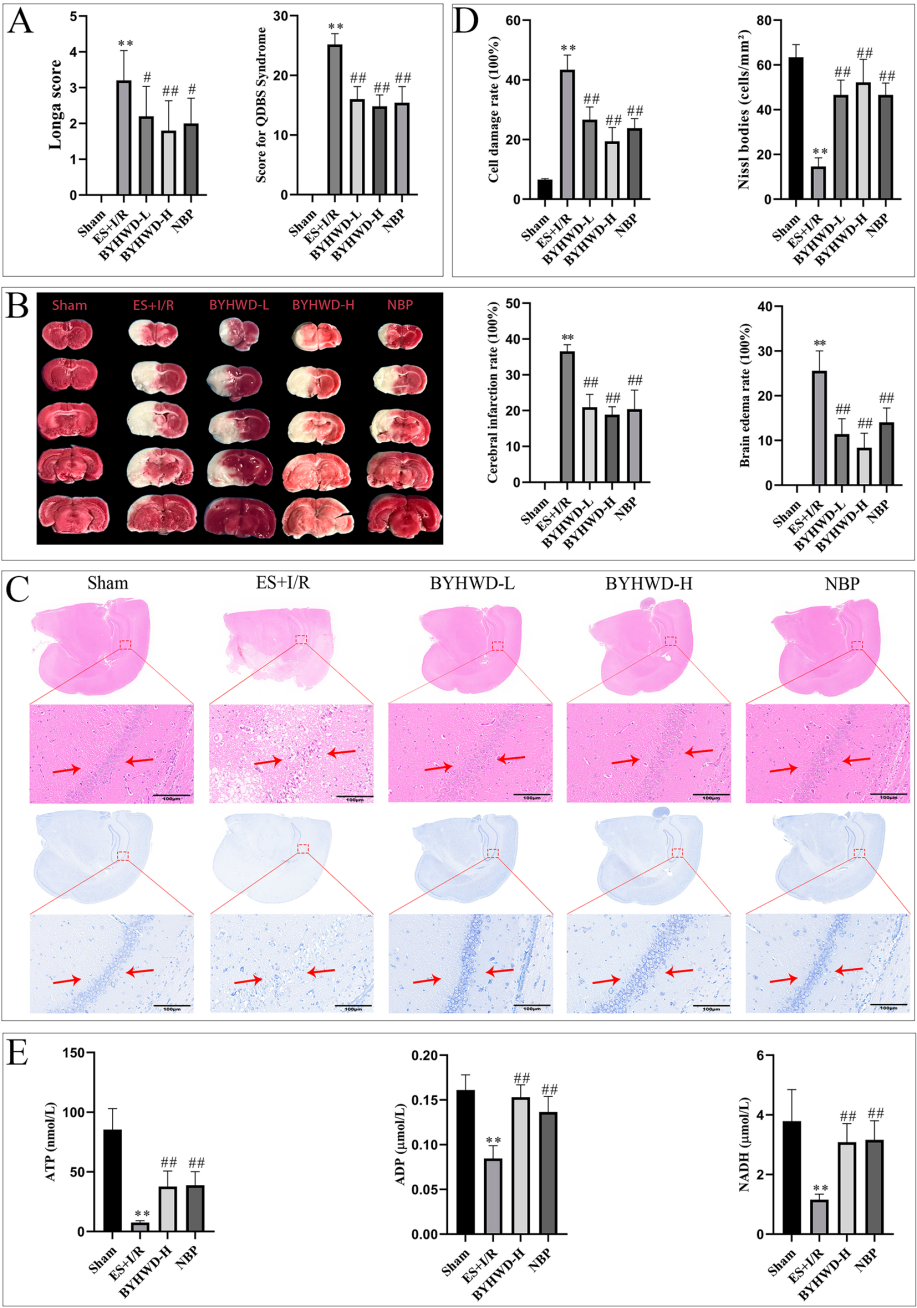
Effects of BYHWD on neurological deficits, Qi deficiency and blood stasis syndrome, and pathological brain damage in rats (mean ± SD, *n* = 5). **A** Comparison of Longa and Qi deficiency and blood stasis scores. **B** TTC staining patterns and comparison of infarct volume and brain edema rate. **C** HE staining and Nissl staining patterns of the hippocampal region (scale bar = 200 μm). **D** Comparison of the rate of cell damage and the number of Nissl bodies in the region of the hippocampus. **E** Comparison of ATP, ADP, and NADH levels in brain tissues among different groups. ^*^P < 0.05, ^**^P < 0.01, vs Sham; ^#^*P* < 0.05, ^##^*P* < 0.01, vs ES + I/R

According to traditional Chinese medicine theory, BYHWD is a foundational formula for treating cerebral infarction with Qi deficiency and blood stasis syndrome. This study employed a rat model of CIRI (established via exhaustive swimming combined with MCAO) to investigate the therapeutic effects of BYHWD. Results demonstrated that compared to the Sham group, the ES + I/R group showed significant increases in Longa scores, Qi deficiency blood stasis syndrome scores, cerebral infarction rate, brain edema rate, and hippocampal neuronal injury rate (*P* < 0.01), alongside a significant reduction in Nissl bodies count (*P* < 0.01). Compared to the ES + I/R group, the BYHWD-L, BYHWD-H, and NBP groups exhibited significantly reduced Longa scores and syndrome scores (*P* < 0.05 or *P* < 0.01), lower cerebral infarction and edema rates (*P* < 0.01), decreased neuronal injury (*P* < 0.01), and increased Nissl bodies count (*P* < 0.01) (Fig. 1A–D).

These results indicated that BYHWD significantly alleviated neurological deficits and pathological brain damage in rats with Qi deficiency blood stasis syndrome following CIRI. The therapeutic effect of BYHWD-H (high-dose) surpassed that of BYHWD-L (low-dose). Therefore, a crude drug concentration of 22.2 g/kg/day was used for subsequent studies.

ATP, ADP, and NADH are critical molecules in cerebral energy metabolism, reflecting the brain’s energy status. Analysis revealed that compared to the Sham group, the ES + I/R group showed significantly decreased levels of ATP, ADP, and NADH in brain tissue (*P* < 0.01). In contrast, the BYHWD-H and NBP groups exhibited significant increases in these energy metabolites (*P* < 0.01) (Fig. 1E), suggesting that BYHWD ameliorates energy metabolism dysfunction in the brain after CIRI.

### Metabonomic characterization

#### Metabonomic quality control

To clarify the mechanism of BYHWD in improving energy metabolism after CIRI with Qi deficiency and blood stasis syndrome rats, metabonomics was used to identify potential biomarkers and signaling pathways.

**Fig. 2:**
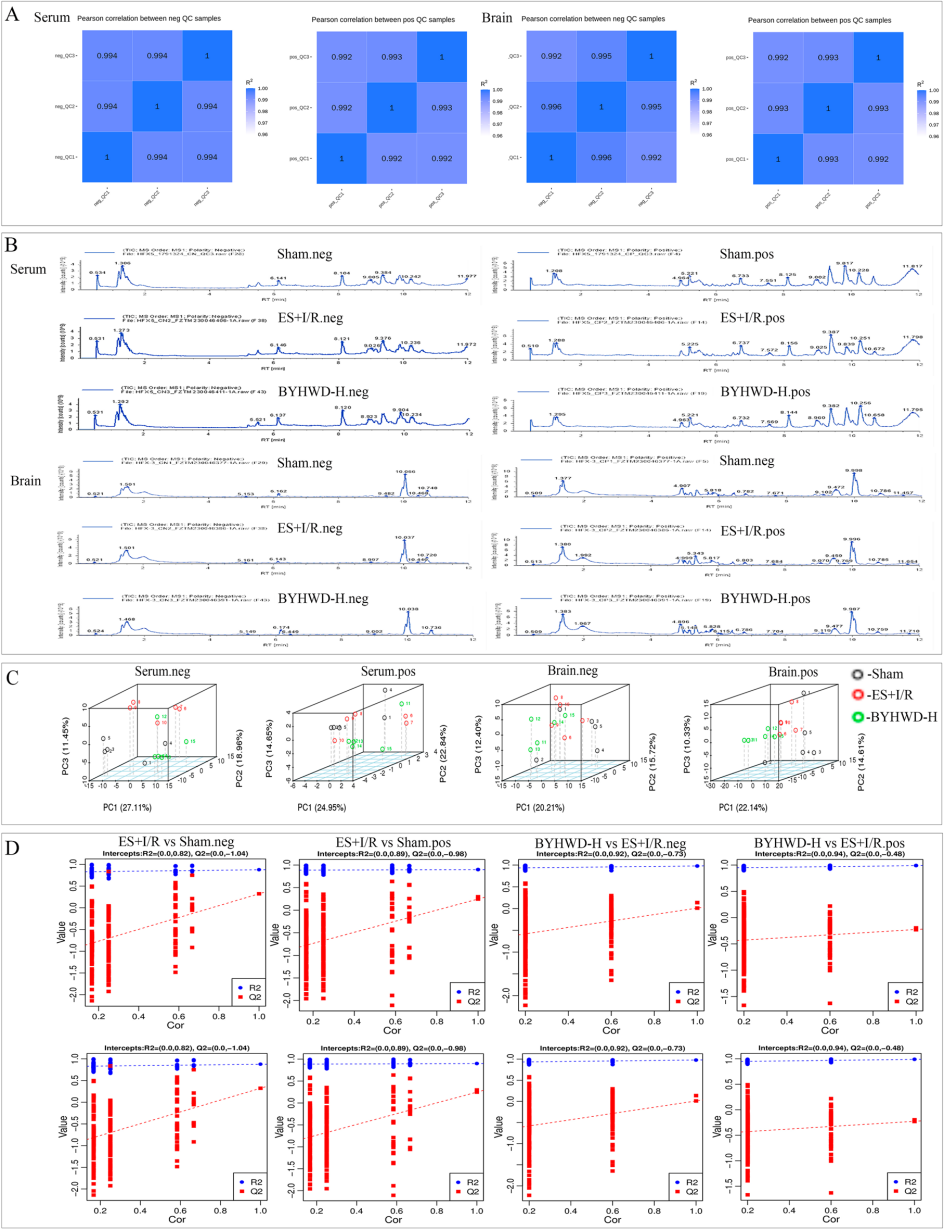
Metabolomics QC sample plots and TIC plots of each group. **A** Correlation analysis of serum and brain tissue untargeted metabolomics QC samples in positive and negative ion mode (*n* = 3). **B** TIC plots of some samples of serum and brain tissue untargeted metabolomics Sham group, ES + I/R group and BYHWD-H group in positive and negative ion mode (*n* = 5). **C** Three-dimensional maps of PCA of serum and brain tissue metabolomics under positive and negative ion mode (confidence interval = 95%) to analyze the metabolite differences between the two groups. **D** PLS-DA replacement test plots of serum and brain tissue metabolomics in the Sham group versus the ES + I/R group, and the BYHWD-H group versus the ES + I/R group under positive and negative ion model, and analysis of whether the PLS-DA model constructs of the groups were overfitted

In this experiment, QC samples were made by mixing all samples equally. This ensured the QC samples' composition closely matched the biological samples, reflecting experimental stability and reproducibility (Fig. 2A). Results showed serum and brain tissue QC samples had a correlation coefficient (R^2^) over 0.99, indicating good stability and reproducibility. Total ion chromatograms (TIC) analyzed intragroup and intergroup differences. Sham, ES + I/R, and BYHWD-H groups had similar intragroup sample peak heights and areas, indicating small intragroup differences and accurate sampling. However, differences in peak heights and intensities existed between Sham and ES + I/R groups, as well as between ES + I/R and BYHWD-H groups, showing intergroup differences (Fig. 2B).

#### PCA and PLS-DA discriminant analysis

PCA is an unsupervised modeling analysis method that can more realistically reflect intergroup differences as well as identify intragroup variation. We used the PCA method to calculate the dimensionality reduction of the data from three groups of serum and brain tissue metabolomics, with the black dots for the Sham group, the red dots for the ES + I/R group, and the green dots for the BYHWD-H group, and the three-dimensional plots of PCA showed that there was no obvious intersection and overlap of samples from the Sham group with the ES + I/R group, and from the ES + I/R group with the BYHWD-H group, suggesting that there was a difference in the metabolites among the groups (Fig. 2C). PLS-DA is a supervised discriminant analysis statistical method that models the relationship between metabolite expression and sample category. In order to verify the reliability of the model, we used the permutation test to verify whether the PLS-DA model is overfitted, and Q^2^ represents the prediction rate of the current model, which indicates the prediction ability of the current model. The results showed that the intercept of the Q^2^ regression line of the PLS-DA model in the vertical coordinate was less than 1 when the Sham group was compared with the ES + I/R group and the ES + I/R group with the BYHWD-H group, suggesting that the predictive model was not overfitting and the model predictive ability was accurate, and that the VIP values obtained based on the PLS-DA model could be used in the subsequent screening of differential metabolites (Fig. 2D).

#### Differential metabolite screening and metabolic pathway enrichment

We analyzed the differential metabolites and their metabolic pathways. A total of 78 differential metabolites were screened in the Sham group compared with the serum samples of the ES + I/R group, of which 21 were up-regulated and 57 were down-regulated, and a total of 24 metabolic pathways were enriched, including arginine biosynthesis, tryptophan metabolism, amino sugar and nucleotide sugar metabolism, insulin resistance, vitamin B6 metabolism, etc. A total of 71 differential metabolites were screened in the BYHWD-H group compared with the serum samples of the ES + I/R group, of which 24 were up-regulated and 47 were down-regulated. A total of 43 metabolic pathways were enriched, including nicotinate and nicotinamide metabolism, AMPK signaling pathway, galactose metabolism, pantothenate and CoA biosynthesis, starch and sucrose metabolism, pentose and glucuronate interconversions, pentose phosphate pathway, taurine and hypotaurine metabolism, the citrate cycle (TCA cycle), glucagon signaling pathway, insulin resistance, etc.

**Fig. 3:**
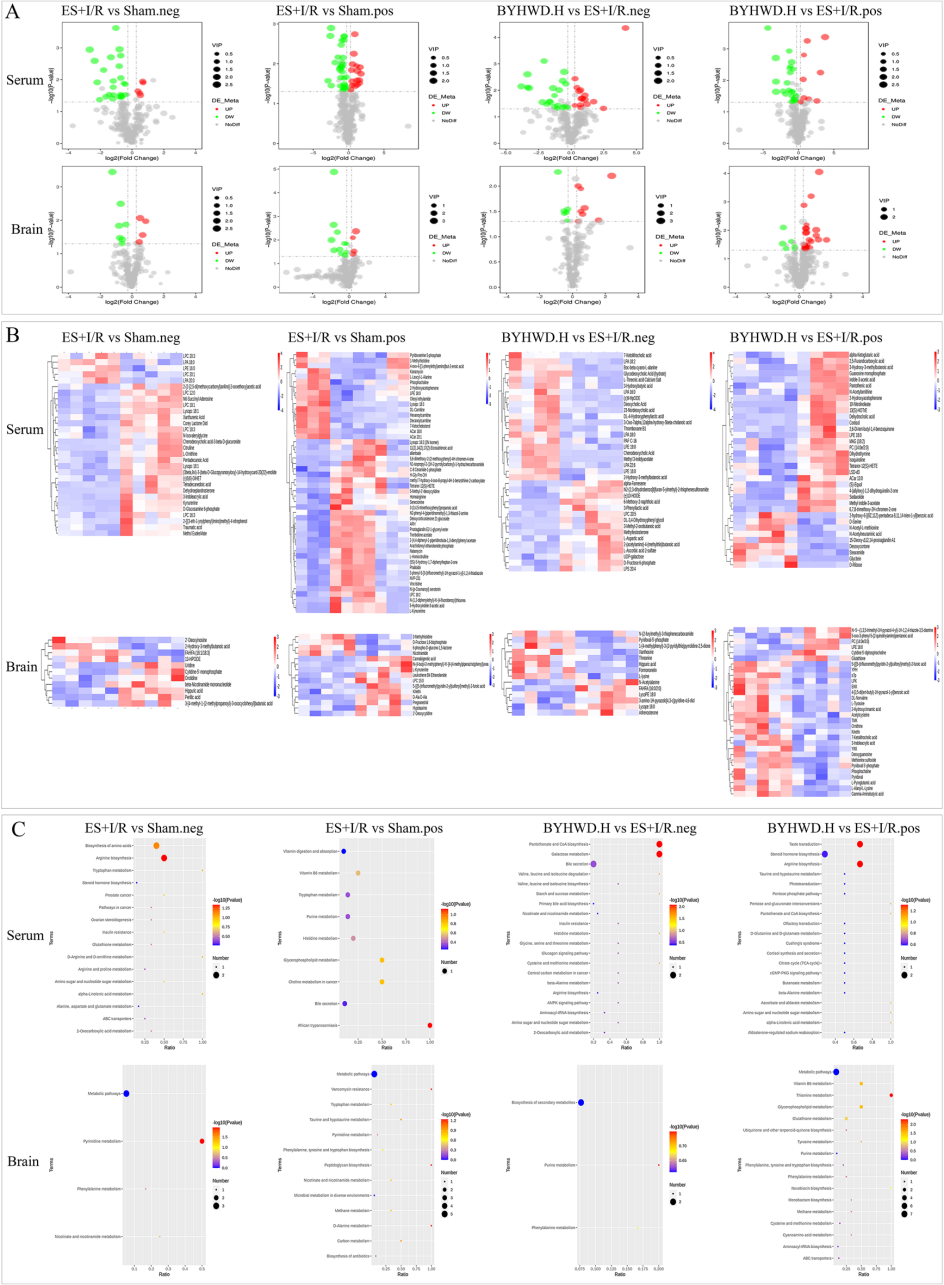
Analysis of differential metabolites and metabolic pathways in serum and brain tissue metabolomics under positive and negative ion patterns (*n* = 5). **A** Volcano plots of differential metabolites in serum and brain tissues under positive and negative ion modes in each group to analyze the overall distribution of differential metabolites. **B** Heat maps of serum and brain tissue differential metabolites in positive and negative ion modes in each group to analyze the differential metabolites and their correlation. **C** KEGG pathway maps of serum and brain tissue in positive and negative ion modes in each group to analyze the different metabolic pathways between the two groups

A total of 26 differential metabolites were screened in the Sham group compared with brain tissue samples from the ES + I/R group, of which 8 were up-regulated and 18 were down-regulated, and a total of 13 metabolic pathways were enriched, including nicotinate and nicotinamide metabolism, phenylalanine metabolism, taurine and hypotaurine metabolism, carbon metabolism, tryptophan metabolism, phenylalanine, tyrosine and tryptophan biosynthesis, etc. BYHWD-H versus ES + I/R group comparison of brain tissue samples screened a total of 44 differential metabolites, of which 32 were up-regulated and 12 were down-regulated. A total of 16 metabolic pathways were enriched in brain tissue samples, including thiamine metabolism, vitamin B6 metabolism, tyrosine metabolism, ubiquinone and other terpenoid-quinone biosynthesis, phenylalanine metabolism, and phenylalanine, tyrosine and tryptophan biosynthesis (Fig. 3).

Brain tissue primarily depends on glucose metabolism for energy, with glycolysis and the TCA cycle being the main pathways. Metabonomic results indicated that pathways such as nicotinate and nicotinamide metabolism, AMPK signaling, galactose metabolism, starch and sucrose metabolism, pentose phosphate pathway, insulin resistance, TCA cycle, taurine and hypotaurine metabolism, thiamine metabolism, tyrosine metabolism, and ubiquinone and other terpenoid-quinone biosynthesis were closely related to glycolysis or the TCA cycle.

Notably, nicotinate and nicotinamide metabolism was enriched in both serum and brain tissue metabolomics. Nicotinamide, the primary metabolite of niacin, is converted to NAD^+^ via NAMPT. NAD^+^ acts as a coenzyme in numerous enzymatic reactions, enhancing energy metabolism by improving glycolysis efficiency and facilitating H^+^ transfer to link the TCA cycle with the respiratory chain [28, 29]. Studies demonstrate that NAD^+^ further promotes AMPK activation by upregulating SIRT1 activity. Activated AMPK enhances glucose uptake, boosts glycolysis efficiency, and strengthens TCA cycle function, thereby maintaining cellular energy homeostasis [30–32]. Therefore, subsequent experiments in this study employed in vivo animal models and in vitro cell models to investigate the mechanism by which BYHWD regulates energy metabolism through modulation of the SIRT1/AMPK signaling pathway.

### BYHWD enhances the activities of key rate-limiting enzymes in glycolysis, key rate-limiting enzymes in the TCA Cycle, and mitochondrial respiratory chain complexes I-IV in brain tissue

In metabolomic studies, we observed that BYHWD ameliorates glycolysis, the TCA cycle, and oxidative phosphorylation in brain tissue following CIRI with Qi deficiency and blood stasis syndrome model. To further investigate these effects, we measured the activities of key rate-limiting enzymes in glycolysis (HK, PFK, and PK), key rate-limiting enzymes in the TCA cycle (CS, α-KGDH, NAD-IDH), and the activities of mitochondrial respiratory chain complexes I-IV.

**Fig. 4:**
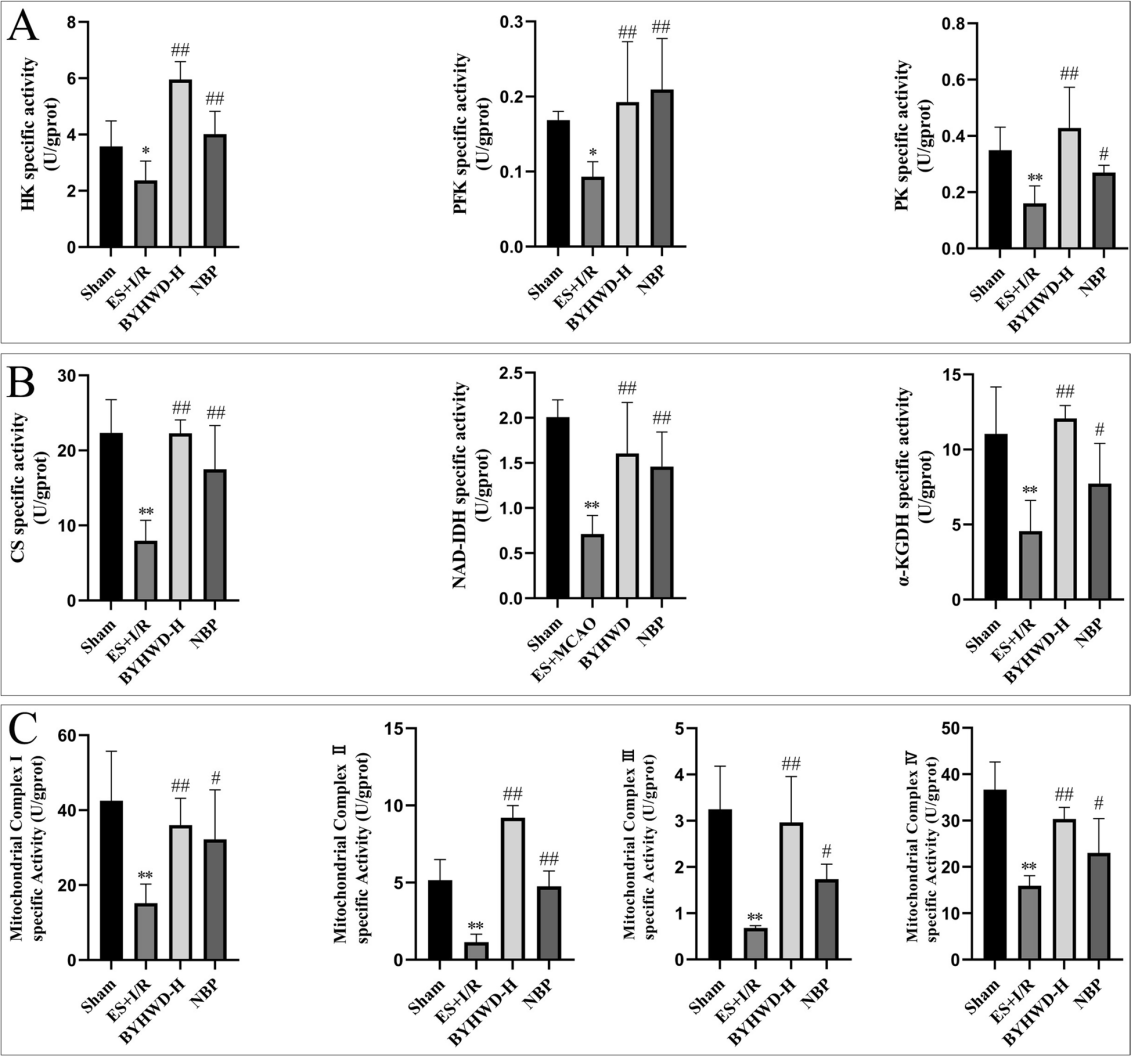
Effects of BYHWD on the activities of key rate-limiting enzymes in glycolysis, TCA cycle, and mitochondrial respiratory chain complexes I–IV (mean ± SD, *n* = 5). **A** Comparison of activities of glycolytic rate-limiting enzymes HK, PFK, and PK. **B** Comparison of activities of TCA cycle rate-limiting enzymes CS, NAD-IDH, and α-KGDH. **C** Comparison of activities of mitochondrial respiratory chain complexes I–IV. ^*^P < 0.05, ^**^P < 0.01, vs Sham; ^#^*P* < 0.05, ^##^*P* < 0.01, vs ES + I/R

Glycolysis is a critical pathway for energy metabolism in brain tissue and plays a role in maintaining energy homeostasis following CIRI. Our results demonstrated that compared to the Sham group, the activities of key rate-limiting enzymes in glycolysis (HK, PFK, and PK) were significantly reduced in the ES + I/R group (*P* < 0.01 and *P* < 0.05). This suggested that the modeling method involving exhaustive swimming followed by MCAO may have suppressed glycolysis, leading to impaired energy metabolism in brain tissue and an inability to effectively respond to CIRI. In contrast, BYHWD treatment significantly increased the activities of HK, PFK, and PK in brain tissue (*P* < 0.01), indicating that BYHWD may enhance glycolysis to restore energy supply in brain tissue (Fig. 4A).

The TCA cycle and oxidative phosphorylation are central processes in cerebral energy metabolism, providing the majority of ATP required to sustain the high-energy demands of neural cells. Compared to the Sham group, the activities of key rate-limiting enzymes in the TCA cycle (CS, NAD-IDH, and α-OGDH) and mitochondrial respiratory chain complexes I-IV were significantly reduced in the ES + I/R group (*P* < 0.01). This implied that the ES + I/R modeling method inhibited the TCA cycle and oxidative phosphorylation, leading to metabolic dysfunction in brain tissue and subsequent impairment of neural cell function and survival. Following BYHWD intervention, the activities of TCA cycle key rate-limiting enzymes (CS, NAD-IDH, and α-OGDH) and mitochondrial respiratory chain complexes I-IV were significantly elevated (*P* < 0.01), suggesting that BYHWD may enhance energy metabolic efficiency and restore ATP generation capacity by activating the TCA cycle and oxidative phosphorylation, thereby improving post-ischemic energy metabolism in brain tissue (Fig. 4B, C).

These findings indicated that BYHWD may have improved energy metabolic disorders in brain tissue following CIRI by enhancing glycolysis and TCA cycle function, increasing mitochondrial respiratory chain complex activities, and restoring impaired energy metabolism.

### BYHWD can promote the expression of proteins related to glycolysis and TCA cycle by regulating SIRT1/AMPK signaling pathway in brain tissue

**Fig. 5:**
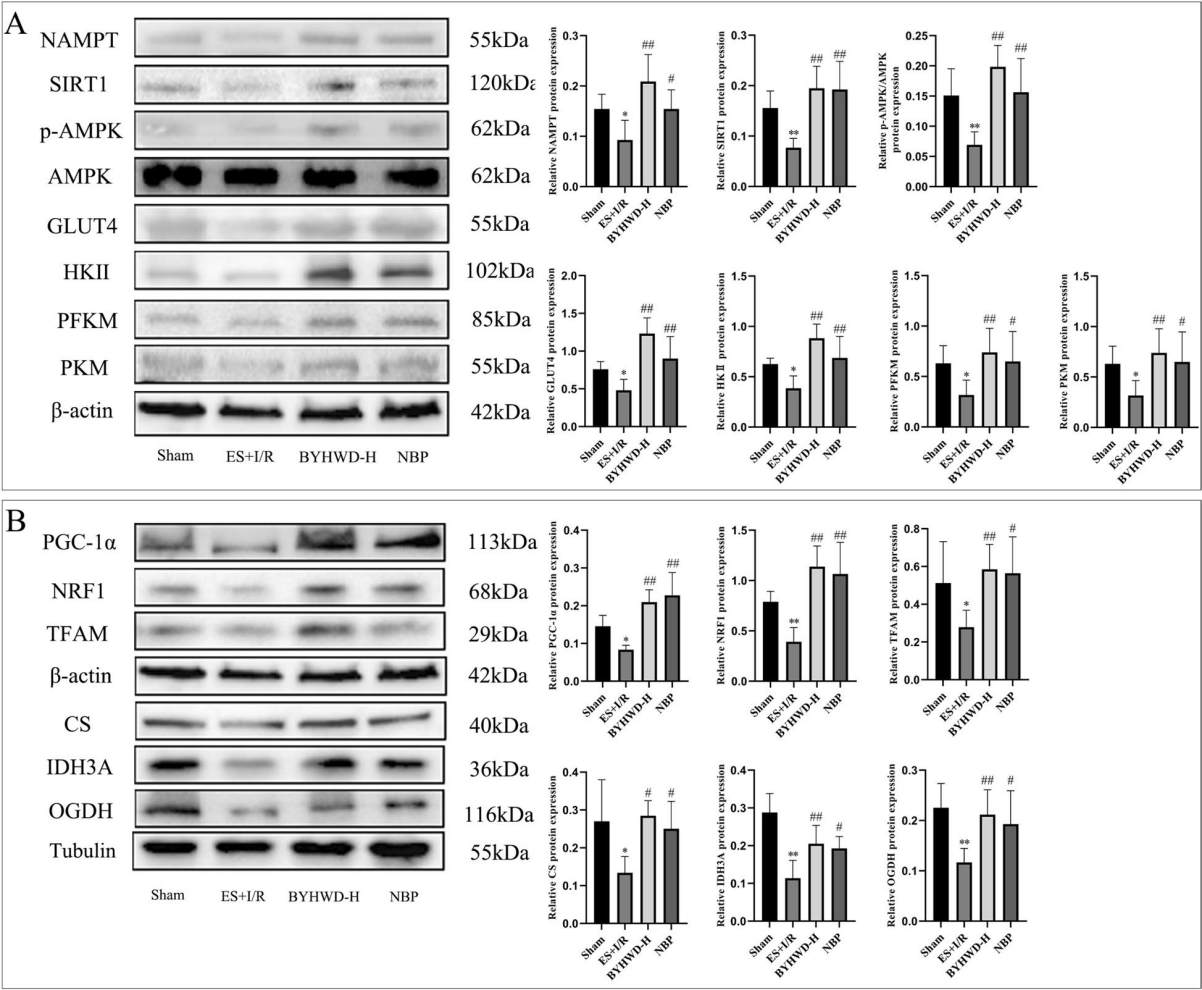
Effects of BYHWD on the expression of proteins related to the SIRT1/AMPK signaling pathway, GLUT4, glycolysis, mitochondrial biogenesis, and the TCA cycle in cerebral tissue (mean ± SD, *n* = 5). **A** Comparison of protein expression levels of the SIRT1/AMPK signaling pathway, GLUT4, and key rate-limiting enzymes of glycolysis among groups. **B** Comparison of protein expression levels related to mitochondrial biogenesis and key rate-limiting enzymes of TCA cycle across groups. ^*^P < 0.05, ^**^P < 0.01, vs Sham; ^#^*P* < 0.05, ^##^*P* < 0.01, vs ES + I/R

The energy supply of the brain is highly dependent on glucose, which is mainly derived from the blood and crosses the blood–brain barrier into the brain tissue with the assistance of glucose transport proteins to participate in the energy metabolism of the brain tissue. In metabolomics, we found that BYHWD could significantly improve the energy metabolism of brain tissues after CIRI with Qi deficiency and blood stasis syndrome by regulating several pathways, including nicotinate and nicotinamide metabolism, and the underlying mechanism may be related to the regulation of the SIRT1/AMPK signaling pathway, which in turn promotes glycolysis and the TCA cycle. As the key regulator of cellular energy metabolism, AMPK is activated under the state of energy stress. Activated AMPK significantly upregulates GLUT4 protein expression, thereby promoting glucose uptake. By enhancing the activity of glycolytic rate-limiting enzymes (e.g., HKII, PFKM) [33, 34] and modulating the TCA cycle [35], AMPK augments glycolytic flux and TCA cycle capacity, subsequently improving cellular energy metabolism efficiency. In addition, both SIRT1 and activated AMPK can maintain normal mitochondrial function by promoting mitochondrial biosynthesis [36–38]. Mitochondria are important sites for the TCA cycle as well as ATP synthesis in cells, and maintaining mitochondrial biosynthesis and function are important for improving the TCA cycle and promoting energy metabolism in brain tissue. Based on the above metabolomics results, in this study, we used Western blot technology to verify the mechanism of BYHWD in improving energy metabolism disorders from the levels of SIRT1/AMPK signaling pathway, GLUT4, glycolysis, mitochondrial biosynthesis, and the expression of TCA cycle-related proteins. Results showed (Fig. 5A, B) that compared to the Sham group, the ES + I/R group exhibited significantly reduced protein expression of NAMPT, SIRT1, p-AMPK, GLUT4, HKII, PFKM, PKM, PGC-1α, NRF1, TFAM, CS, IDH3A, and OGDH (*P* < 0.01 and *P* < 0.05), indicating that the ES + I/R model markedly suppressed the SIRT1/AMPK signaling pathway, glycolysis, and the TCA cycle, thereby inducing cerebral energy metabolism dysfunction. Following BYHWD intervention, the expression levels of these proteins in cerebral tissue were significantly elevated (*P* < 0.01 and *P* < 0.05). These findings aligned with metabolomics data, indicating that BYHWD improved cerebral energy metabolism dysfunction by modulating the SIRT1/AMPK signaling pathway to enhance glucose uptake and metabolism, sustain mitochondrial function, and augment glycolysis and TCA cycle capacity.

### In vitro cell experiments showed that BYHWD-containing serum improved the viability and energy metabolism of neurons after OGD/R injury.

**Fig. 6:**
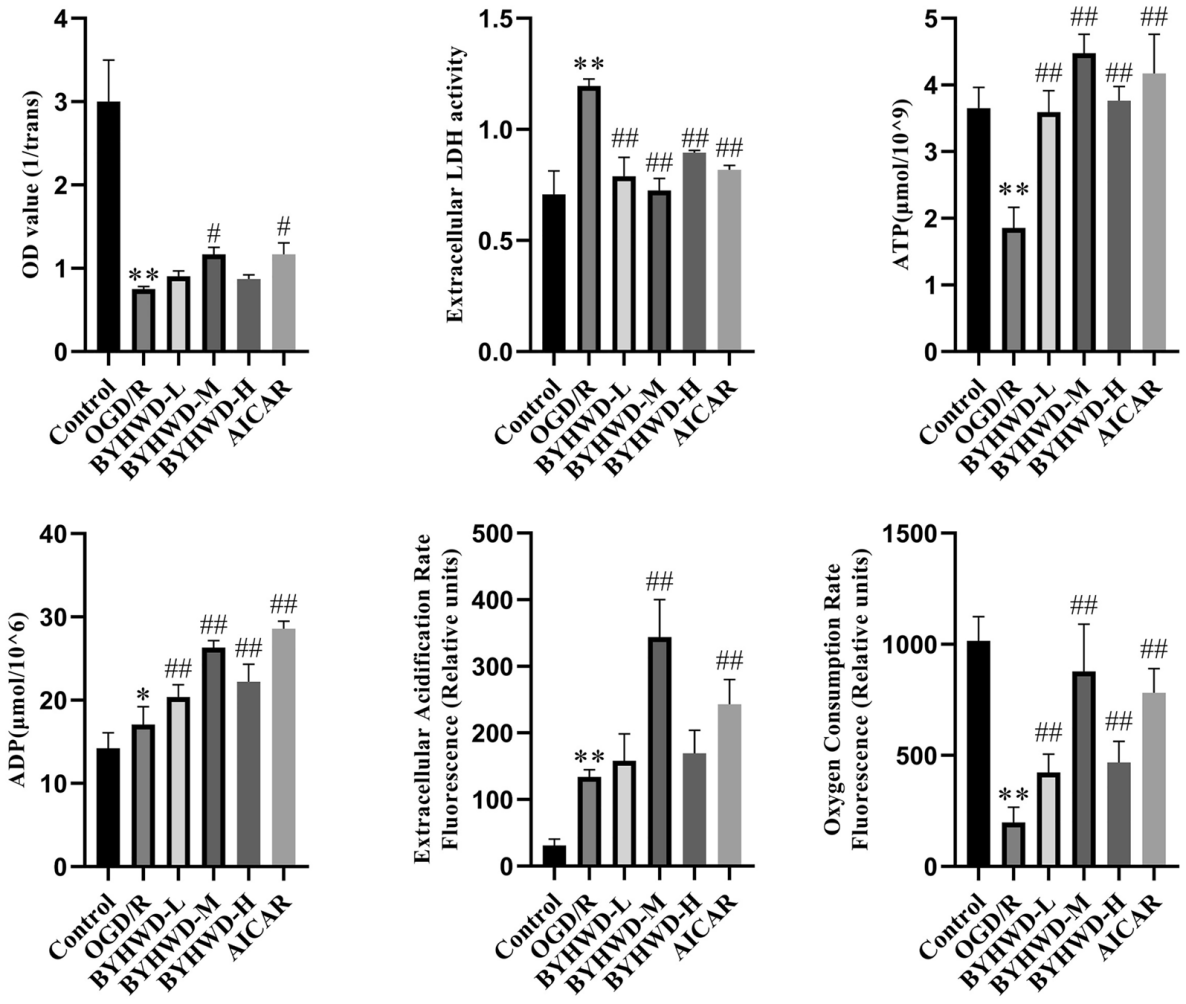
Effect of BYHWD on HT22 neuronal cell viability, LDH activity and energy metabolism after OGD/R (mean ± SD, *n* = 5). ^*^P < 0.05, ^**^P < 0.01, vs Sham; ^#^*P* < 0.05, ^##^*P* < 0.01, vs OGD/R

To further verify whether BYHWD activates the SIRT1/AMPK signaling pathway, thereby improving glycolysis and TCA cycle function, and alleviating cerebral energy metabolic disorders, this study used HT22 cells to establish an OGD/R model of neuronal injury to explore the mechanisms of BYHWD. Cell viability and LDH activity were measured by CCK-8 assay and colorimetry to evaluate the protective effect of BYHWD against OGD/R injury. The results showed (Fig. 6) that compared to the Control group, cell viability was significantly reduced, and LDH activity in the cell supernatant was increased in the OGD/R group, indicating cell damage after OGD/R (*P* < 0.01). Compared to the OGD/R group, cell viability was significantly increased in the BYHWD-M and AICAR groups (*P* < 0.05), while no significant difference was found between the BYHWD-L and BYHWD-H groups. Additionally, LDH activity in the cell culture supernatant was significantly decreased in the BYHWD-L, BYHWD-M, BYHWD-H, and AICAR groups compared to the OGD/R group (*P* < 0.01). These results suggested that BYHWD-containing serum alleviated OGD/R-induced cell damage and promoted cell survival.

ATP, the primary cellular energy carrier, is hydrolyzed to ADP and inorganic phosphate to release energy for neuronal functions such as neurotransmission, neurotransmitter release, and ion pump activity [39]. Extracellular acidification rate (ECAR) and oxygen consumption rate (OCR) are key indicators of cellular glycolysis and the TCA cycle, reflecting glycolytic and TCA cycle capacities. To further investigate the effects of BYHWD on cellular energy metabolism, we measured ATP, ADP levels, and used ECAR and OCR to assess glycolysis and TCA cycle function. The results showed (Fig. 6) that compared to the Control group, ATP levels and OCR were significantly reduced, while ADP levels and ECAR were significantly increased in the OGD/R group (*P* < 0.01 and *P* < 0.05). These changes indicated impaired TCA cycle function and compensatory enhanced glycolysis after OGD/R, but overall reduced energy metabolism. Compared to the OGD/R group, ATP and ADP levels and OCR were significantly increased in the BYHWD-L, BYHWD-M, BYHWD-H, and AICAR groups (*P* < 0.01), and ECAR was significantly increased in the BYHWD-M and AICAR groups (*P* < 0.01). This suggested that BYHWD enhanced glycolysis and TCA cycle capacity in neurons after OGD/R, thereby promoting cellular energy metabolism.

### BYHWD upregulates the SIRT1/AMPK signaling pathway and promotes the expression of proteins related to glycolysis and the TCA cycle in neurons following OGD/R injury

**Fig. 7:**
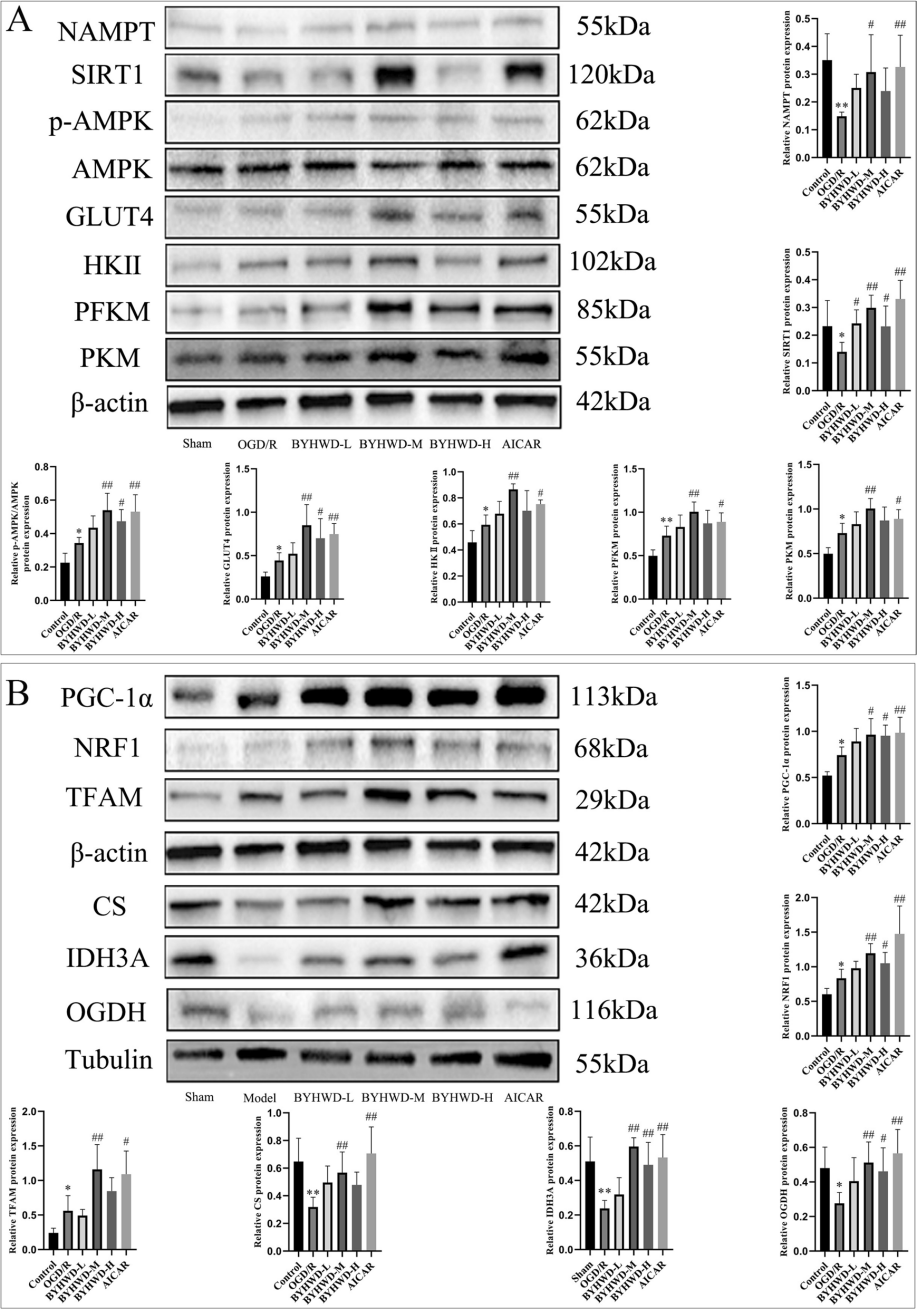
Effects of BYHWD on the protein expression related to the SIRT1/AMPK signaling pathway, GLUT4, glycolysis, mitochondrial biogenesis, and the TCA cycle in neurons after OGD/R injury (mean ± SD, *n* = 5). **A** Comparison of protein expression related to the SIRT1/AMPK signaling pathway, GLUT4, and key rate-limiting enzymes in glycolysis across different groups. **B** Comparison of protein expression related to mitochondrial biogenesis and key rate-limiting enzymes in the TCA cycle across different groups. ^*^P < 0.05, ^**^P < 0.01, vs Sham; ^#^*P* < 0.05, ^##^*P* < 0.01, vs OGD/R

To further explore the mechanism by which BYHWD regulates energy metabolism in neurons, we examined the protein expression of SIRT1/AMPK signaling pathway-related proteins and key rate-limiting enzymes in glycolysis and the TCA cycle. Western blot results (Fig. 7A, B) showed that compared to the Control group, the protein expression of NAMPT and SIRT1 was significantly reduced in the OGD/R group (*P* < 0.01 and *P* < 0.05), while p-AMPK, GLUT4, HKII, PFKM, PKM, PGC-1α, NRF1, and TFAM were significantly increased (*P* < 0.01 and *P* < 0.05). Additionally, the protein expression of CS, IDH3A, and OGDH was significantly decreased (*P* < 0.01 and *P* < 0.05). Despite the reduction in NAMPT and SIRT1 protein expression in the OGD/R group, p-AMPK protein was still significantly upregulated, possibly due to an adaptive response to OGD/R. During ischemia and hypoxia, low-energy states stimulate AMPK activation, which promotes glycolysis and mitochondrial biogenesis to meet cellular energy demands. However, ischemic and hypoxic cellular environments are complex, and factors such as increased oxidative stress and mitochondrial dysfunction limit the effectiveness of this regulatory mechanism in restoring metabolic disorders. Compared to the OGD/R group, the protein expression of NAMPT, SIRT1, p-AMPK, GLUT4, HKII, PFKM, PKM, PGC-1α, NRF1, TFAM, CS, IDH3A, and OGDH was significantly increased in the BYHWD-M and AICAR groups (*P* < 0.01 and *P* < 0.05). This suggested that BYHWD may have enhanced NAMPT expression to promote NAD⁺ synthesis, thereby activating the SIRT1/AMPK signaling pathway. This activation increased glucose uptake and mitochondrial biogenesis, improved glycolysis and TCA cycle capacity, and alleviated energy metabolic disorders following OGD/R injury, restoring cellular energy metabolism.

## Discussion

With the acceleration of population aging, cerebral infarction has become the second leading fatal disease globally, making its prevention and treatment an urgent priority. In TCM, differentiation of syndromes and treatment based on syndrome differentiation has shown significant efficacy in the treatment of cerebral infarction. TCM posits that the onset of cerebral infarction is closely related to Qi and blood, with Qi deficiency and blood stasis being a pivotal pathomechanism. Prolonged high-intensity exercise or physical frailty can lead to Qi deficiency. When Qi is deficient, it fails to propel blood circulation effectively, resulting in blood stasis. This stasis obstructs the brain collateral channels and manifests as cerebral infarction. However, the absence of animal models that integrate disease and syndrome has greatly impeded the study of TCM in the prevention and treatment of cerebral infarction. In this experiment, we based on this TCM theory and previous studies by other researchers, we first established a Qi deficiency model in rats through continuous exhaustive swimming. Subsequently, we combined this with MCAO to develop a disease-syndrome integrated model of Qi deficiency and blood stasis-induced CIRI. The model's validity was assessed by evaluating various indicators of Qi deficiency and blood stasis in the rats, such as their mental state, coat condition, food intake, tongue appearance, and eye color. The results demonstrated that this model closely aligns with TCM's theoretical understanding of cerebral infarction with Qi deficiency and blood stasis. It provides a more accurate and practical model for the pathological processes involved, thereby offering a valuable tool for studying the prevention and treatment of cerebral infarction using TCM. Moreover, our study also confirmed that this model is characterized by more severe pathological damage to brain tissue, more pronounced energy metabolism disorders, and a more severe inflammatory response [40]. BYHWD, a classic traditional Chinese medicine formula for cerebral infarction, has demonstrated significant clinical efficacy in practice [41, 42]. In this study, we established a rat model of CIRI with Qi deficiency and blood stasis syndrome using exhaustive swimming combined with MCAO. Results showed that rats with Qi deficiency and blood stasis syndrome exhibited severe neurological deficits, Qi deficiency and blood stasis symptoms, and cerebral pathological damage. BYHWD intervention ameliorated these impairments to varying degrees, further confirming its therapeutic potential against CIRI.

Energy metabolism dysfunction is recognized as the initiating factor in CIRI. During ischemia, rapid ATP depletion due to energy substrate deficiency reduces Na⁺-K⁺-ATPase and Ca^2^⁺-ATPase activities, leading to intracellular Ca^2^⁺ overload, neuronal edema, and structural damage [43–45]. Concurrently, glucose-oxygen deprivation impairs mitochondrial respiratory chain complexes I–IV [46], prolonging energy deficits and triggering metabolic chaos. Persistent ischemia promotes mitochondrial permeability transition pore opening, releasing reactive oxygen species and cytochrome C, which exacerbate cerebral injury [47–49]. ATP, ADP, and NADH are critical indicators of energy metabolism [50]. In our experiments, rats in the ES + I/R group showed significantly reduced levels of ATP, ADP, and NADH in brain tissue, indicating severe energy metabolic disorders. However, BYHWD treatment markedly restored these metabolites, suggesting its neuroprotective effects via enhanced energy metabolism.

Although experimental evidence has demonstrated BYHWD improves cerebral energy metabolism following CIRI with Qi deficiency and blood stasis syndrome, its precise targets and mechanisms remain insufficiently elucidated. Metabolomics, a key component of systems biology, investigates the mechanisms of life processes by analyzing the involvement of differential metabolites [51]. Metabolomics offers precise and direct phenotypic insights into biological systems, enabling rapid identification of pathology- or therapy-relevant metabolites and metabolic pathways. This technique holds significant promise for deciphering the mechanisms of TCM in treating complex diseases [52, 53]. Therefore, in this study, we used metabolomics to analyze the metabolic changes in the serum and brain tissue of rats in the Sham group, the ES + I/R group, and the BYHWD-H group. The results showed that the comparison among these groups revealed multiple metabolites and signaling pathways related to energy metabolism. The main biological processes involved were glycolysis and the TCA cycle. This indicated that there were significant energy metabolic changes in rats with cerebral ischemia reperfusion injury and Qi deficiency and blood stasis syndrome. BYHWD may have improved energy metabolic disorders in brain tissue by promoting glycolysis and the TCA cycle.

Glycolysis plays a critical role in sustaining cerebral energy metabolism under ischemic-hypoxic conditions. Following cerebral infarction, glycolysis generates ATP through glucose breakdown, transiently maintaining metabolic activity in brain tissue [54, 55]. Our metabolomic findings revealed a significant enrichment in glycolysis-related pathways. Galactose, a key component of brain phospholipids in neural systems, participates in glucose metabolism. It is metabolized via the galacturonic acid pathway to produce glucose-6-phosphate (G6P), which then enters the glycolytic pathway [56]. Starch and sucrose metabolism begins with d-fructose, where reversible reactions mediated by maltodextrin glucosidase yield critical intermediates such as G6P and fructose-6-phosphate (F6P), interacting dynamically with glycolysis [57]. Pentose and glucuronate, two carbohydrate metabolites closely linked to energy metabolism, are primarily processed through the pentose phosphate pathway. This pathway intersects with glycolysis by generating F6P and glyceraldehyde-3-phosphate, thereby fueling glycolytic flux [58]. AMPK, a central regulator of bioenergetics, is activated under glucose or ATP deprivation. Activated AMPK enhances cellular energy production by upregulating glucose uptake and glycolysis [59, 60], while suppressing energy-consuming processes to maintain metabolic homeostasis. Additionally, carbon metabolism, insulin, and glucagon modulate glycolysis through diverse mechanisms [61–63].

The TCA cycle is the primary mechanism for energy acquisition in organisms. Our study identified a significant enrichment in the TCA cycle and multiple closely associated signaling pathways. Thiamine (vitamin B1) is converted to thiamine diphosphate in vivo, which participates in acetyl-CoA synthesis and TCA cycle enzymatic reactions, thereby promoting ATP production and maintaining normal neurological function [64, 65]. Ubiquinone (coenzyme Q10), predominantly localized in the mitochondrial inner membrane, serves as a coenzyme for multiple TCA cycle enzymes and a critical component in rate-limiting reactions of the mitochondrial respiratory chain [66]. Additionally, tyrosine metabolism is intricately linked to the TCA cycle. Tyrosine, an essential ketogenic and gluconeogenic amino acid, is metabolized from phenylalanine via transamination to form p-hydroxyphenylpyruvate. This intermediate is subsequently converted to homogentisate by hydroxyphenylpyruvate hydroxylase, and homogentisate undergoes homogentisate dioxygenase-catalyzed hydrolysis to yield acetyl-CoA, acetoacetate, and fumarate, which enter the TCA cycle and oxidative phosphorylation. Tyrosine also serves as a precursor for catecholamine synthesis via tyrosine hydroxylase-mediated enzymatic reactions, contributing to systemic energy homeostasis [67].

The nicotinate and nicotinamide metabolism plays a pivotal role in energy metabolism, wherein nicotinate and nicotinamide are converted to NAD⁺ via NAMPT. As a key coenzyme, NAD⁺ not only stabilizes cellular metabolism through redox reactions, but also regulates glycolysis, the TCA cycle, and oxidative phosphorylation by activating pathways such as SIRT1 and AMPK, thereby critically sustaining energy homeostasis [31, 68–70]. Building on these findings, we validated the mechanism by which BYHWD modulates glycolysis, mitochondrial biogenesis, and the TCA cycle through the SIRT1/AMPK signaling axis.

Under hypoxic-ischemic conditions, brain tissue maintains cellular energy supply by promoting glycolysis [71]. Key rate-limiting enzymes of glycolysis, such as HK, PFK, and PK, phosphorylate intracellularly absorbed glucose, enhancing glucose utilization and releasing a small amount of energy during this process [72, 73]. After glucose is metabolized via glycolysis to produce pyruvate, it is converted into acetyl-CoA within mitochondria and enters the TCA cycle. Through catalysis by enzymes such as CS, NAD-IDH, and α-KGDH, NADH, flavin adenine dinucleotide, and guanosine triphosphate are generated. Ultimately, large amounts of ATP are produced with the assistance of mitochondrial respiratory chain complexes. Using colorimetric assays, we measured the activities of key rate-limiting enzymes in glycolysis (HK, PFK, PK), the TCA cycle (CS, NAD-IDH, α-KGDH), and mitochondrial respiratory chain complexes I-IV in brain tissue. The results showed that the activities of these enzymes and complexes were significantly reduced in the ES + I/R group of rats. However, BYHWD intervention markedly increased their activities, demonstrating that BYHWD enhances glycolysis and the TCA cycle in brain tissue following CIRI. Additionally, to further clarify the mechanisms of BYHWD, an HT22 cell OGD/R model was used to simulate in vivoCIRI. The study found that a 5% BYHWD-containing serum significantly increased cell viability and reduced cell damage after OGD/R. Moreover, the BYHWD-containing serum significantly increased cellular ATP and ADP levels, as well as ECAR and OCR, further confirming that BYHWD improves energy metabolic disorders by regulating glycolysis and TCA cycle function.

To further elucidate the potential mechanisms by which BYHWD improves energy metabolism after CIRI, our study validated the SIRT1/AMPK signaling pathway and related protein expression levels using Western blot. The results demonstrated that in a rat model of CIRI with Qi deficiency and blood stasis syndrome induced by ES + I/R, the expression of SIRT1/AMPK pathway-related proteins (NAMPT, SIRT1, p-AMPK), glycolysis-related proteins (GLUT4, HKII, PFKM, PKM), mitochondrial biogenesis-related proteins (PGC-1α, NRF1, TFAM), and TCA cycle-related proteins (CS, IDH3A, OGDH) in brain tissue were significantly downregulated. However, BYHWD intervention markedly upregulated the expression of these proteins. In vitro, we further investigated the mechanism of BYHWD using an HT22 neuronal cell OGD/R injury model. The results demonstrated that BYHWD-treated HT22 cells exhibited a similar upregulation trend in the expression levels of these proteins, consistent with the in vivo findings. These results confirmed that BYHWD restored energy metabolism in brain tissue post-reperfusion by activating the SIRT1/AMPK signaling pathway, promoting glucose uptake, and enhancing glycolysis and TCA cycle capacity.

## Conclusion

**Fig. 8:**
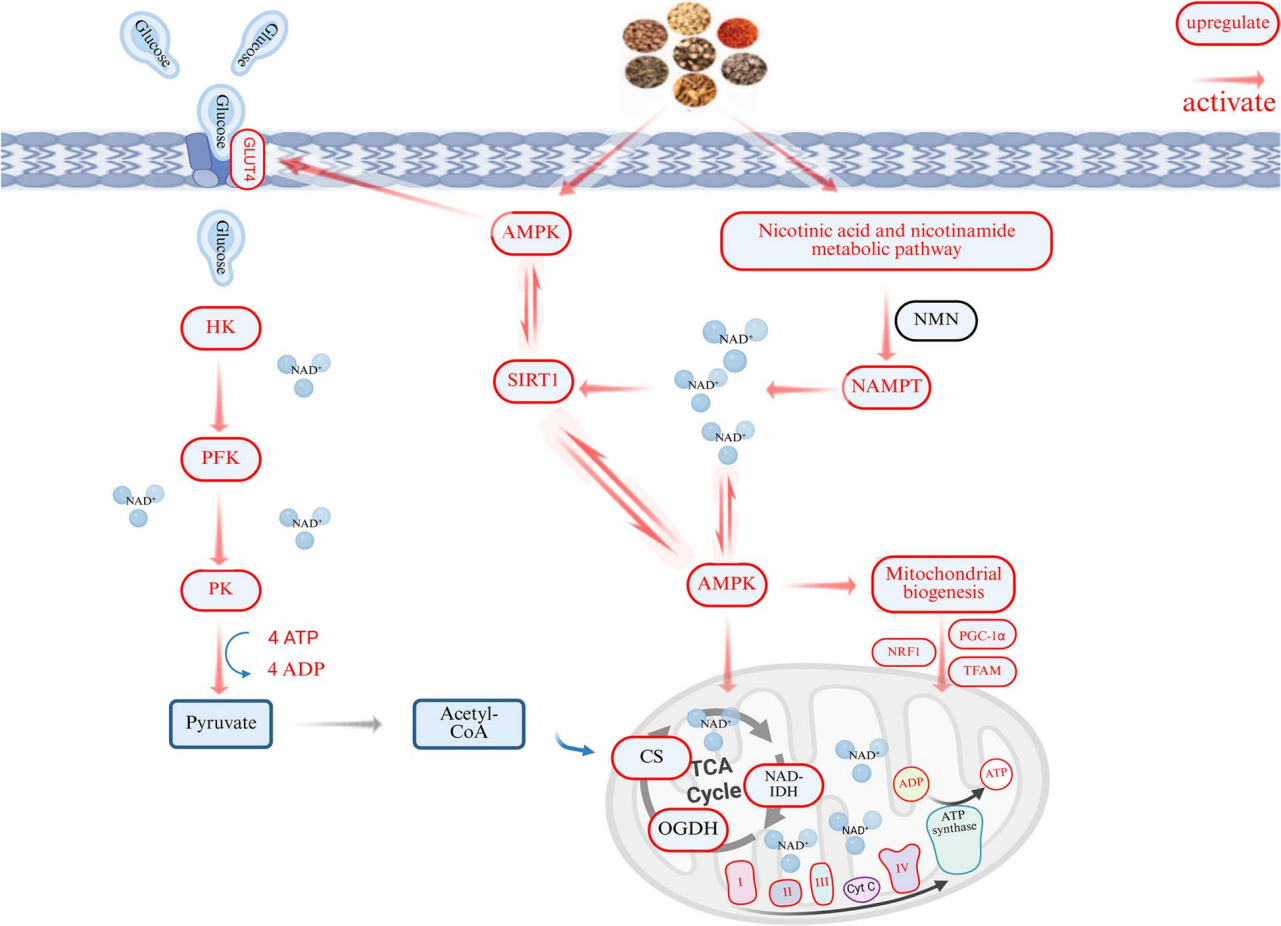
Mechanism diagram of BYHWD. BYHWD improved energy metabolism dysfunction by regulating the SIRT1/AMPK signaling pathway, promoting glucose uptake and mitochondrial biogenesis, and enhancing glycolysis and TCA cycle capacity

In conclusion, this study demonstrated that BYHWD alleviated energy metabolism dysfunction caused by CIRI through regulating the SIRT1/AMPK pathway, increasing glucose uptake, and improving glycolysis and TCA cycle function (Fig. 8). These findings deepen our understanding of the pharmacological mechanisms of BYHWD and provide insights into TCM strategies for preventing and treating cerebral infarction. However, given the complexity of disease pathogenesis and progression, single-omics approaches remain insufficient to fully unravel BYHWD’s therapeutic mechanisms. Future studies will integrate multi-omics technologies to systematically identify therapeutic targets and bioactive components of BYHWD, thereby comprehensively elucidating its anti-ischemic mechanisms.

## Data Availability

The datasets utilized and/or analyzed in this study are available from the corresponding author upon reasonable request.

## Abbreviations

BYHWD: Buyang Huanwu decoction
QDBS: Qi deficiency and blood stasis
CIRI: Cerebral ischemia–reperfusion injury
TCA: Tricarboxylic acid
OGD/R: Oxygen–glucose deprivation/reperfusion
ATP: Adenosine triphosphate
TCM: Traditional Chinese medicine
MCAO: Middle cerebral artery occlusion
ECL: Enhanced chemiluminescence
DMEM: Dulbecco’s modified eagle medium
TTC: 2,3,5-Triphenyltetrazolium chloride
HE: Hematoxylin–eosin
ADP: Adenosine diphosphate
NADH: Nicotinamide adenine dinucleotide
HK: Hexokinase
PFK: Phosphofructokinase
PK: Pyruvate kinase
CS: Citrate synthase
NAD-IDH: Nicotinamide adenine dinucleotide-isocitrate dehydrogenase
α-KGDH: Alpha-ketoglutarate dehydrogenase
ECAR: Extracellular acidification rate
OCR: Oxygen consumption rate
HKII: Hexokinase II
PFKM: Phosphofructokinase, muscle
PKM: Pyruvate kinase, muscle
IDH3A: Isocitrate dehydrogenase 3 alpha
OGDH: Oxoglutarate dehydrogenase
NAMPT: Nicotinamide phosphoribosyltransferase
SIRT1: Sirtuin 1
AMPK: Adenosine 5′-monophosphate-activated protein kinase
GLUT4: Glucose transporter 4
PGC-1α: Peroxisome proliferators-activated receptor γ coactivator l alpha
NRF1: Nuclear respiratory factor 1
TFAM: Mitochondrial transcription factor A
UHPLC-MS/MS: Ultra-high-performance liquid chromatography-tandem mass Spectrometry
QC: Quality control
IOD: Integral optical density
ANOVA: One-way analysis of variance
LSD: Least significant difference
TIC: Total ion chromatograms
PCA: Principal component analysis
PLS-DA: Partial least squares discrimination analysis
NBP: Butylphthalide
AICAR: 5-Aminoimidazole-4-carboxamide ribonucleotide
FBS: Fetal bovine serum
G6P: Glucose-6-phosphate
F6P: Fructose-6-phosphate
